# Ten simple rules for writing a popular science book

**DOI:** 10.1371/journal.pcbi.1005808

**Published:** 2018-02-01

**Authors:** Adam J. Kucharski

**Affiliations:** Centre for the Mathematical Modelling of Infectious Disease, London School of Hygiene and Tropical Medicine, London, United Kingdom; Dassault Systemes BIOVIA, UNITED STATES

Scientists have a long history of writing for a general audience [1]. By communicating ideas and discoveries to the wider public, popular science books can generate debate, influence culture, and inspire future researchers. They can also provide new skills and opportunities, and in some cases a second career, for their authors [2].

Writing scientific papers is a central part of academic life, but there are some notable differences when it comes to publishing books for a wider audience. Based on my experience of writing and contributing to popular science books [3–5], the following rules summarise the main points a budding author needs to consider, from initial proposal through to postrelease publicity.

## Rule 1: Build writing experience

There’s an old adage that to write a good novel, you should write a complete draft, put it in a drawer, then forget about it and start work on what will become the real book. Nonfiction is similar. Writing takes a huge amount of practice, which means it’s a good idea to first build up a portfolio of articles, stories, and blog posts. Not all of these attempts will work out; it’s likely that many of your words will go unpublished. However, writing regularly means you can explore and refine your style. In the process, you’ll develop many of the skills necessary for book writing, from spotting stories and pitching ideas to structuring and editing content. It will also help you decide whether you really want to take on a book project.

## Rule 2: Find the right topic

If you have an idea for a book, there are two main questions you should consider. Why does this book need to be published now? And why are you the person to write it? It helps if the topic is timely and new (or presents a new take on a familiar issue). Try thinking about what’s important in the medium term: your eventual publication date may be three to four years away, so what’s currently in the news may not still be topical. You also need to think about how your academic background relates to the book. Although it might seem easiest to focus on your specific area of research, there are benefits to telling a wider story. First, it’s more likely to be relevant to a general reader, who may be broadly interested in your field but not so keen on a whole book about one subtopic. Second, it can make book writing more interesting for you because you’ll be able to discover new things as you research and write.

## Rule 3: Get an agent

If you want to write a trade book (i.e., for a general audience), it helps to have a literary agent. Although they typically take around a 15% cut of your income, most publishers won't look at a submission unless it comes from a reputable agent, and it’s better to have 85% of a book advance from a good publisher than 100% of nothing. Agents will also edit the book proposal, pitch to publishers, negotiate contracts, advise on potential problems, and handle royalties.

How do you get an agent? It helps if you have a strong book idea and you’ve recently done something that stands out. If you’re an established academic, you may have high-profile discoveries and projects to your name; if you’re more junior, you might have won an award or given talks that have gained a lot of attention. Remember, agents and publishers are looking to sign you as well as the book.

## Rule 4: Write a proposal

Nonfiction books are generally submitted to publishers in the form of a proposal. Like research grants, there are certain conventions that must be followed [6]. Proposals usually consist of about 5,000 words outlining what the book will cover, why you are qualified to write it, and what the competition is. Authors will often submit a sample chapter as well, usually from midway through the book, to give the publisher an idea of their writing style (which means another few thousand words). If this sounds like a lot, spare a thought for fiction writers, who generally have to finish the whole novel first. A good agent will help you edit the proposal to get it in as good a shape as possible. Although you should listen to their advice, it’s important that you’re comfortable with the focus of the book. Don’t add things that you aren’t happy (or qualified) to write about.

## Rule 5: Pitch to publishers

Once the proposal is ready, your agent will send it to dozens of editors at different publishers. This will likely lead to dozens of rejection e-mails. Despite the classic stories of vicious rejections (Knopf famously called Orwell's *Animal Farm* ‘stupid and pointless’ [7]), rejections are often polite and reasonable. Some editors may have recently acquired a book on a similar topic or had a bad run with that genre in the past. Or they may like the idea, but their sales team—who will eventually be pitching the book to retailers—do not.

If an editor is interested, they’ll often get you on the phone to find out more about your plans and give you the chance to find out more about theirs. Then, if all is well, the offers will start coming in. Publishers typically offer an advance against royalties, with the money spread over several payments, e.g., payment on signature, manuscript delivery, hardback, and paperback publication. So, a big headline number may ultimately be split over three or more years. On the plus side, however, there is often potential to sign foreign language deals as well as English ones.

## Rule 6: Find your structure and voice

First person or third person? Narrative driven or explanatory? Lighthearted or serious? Popular science books allow for a much wider range of writing styles than academic papers. In the proposal, you’ll have outlined the overall book structure, but you’ll also need to decide how individual sections and chapters fit together. You might want to follow certain characters or include anecdotes and history to motivate the scientific content. If you can show the conflicts and struggles that shaped the science, it will help draw your readers into the research [8].

Your writing will also have a personality—your ‘voice’ on the page—and this will influence the feel of the book. Some writers have very distinctive, and possibly even distracting, voices. In popular science writing, it’s particularly important to avoid condenscending to the reader. Although they might not know scientific jargon, they should be able to grasp the concepts if you describe them well. As the old media adage goes, never overestimate the knowledge of your readers or underestimate their intelligence [9].

## Rule 7: Research and interview

A typical 250-page popular science book will contain around 75,000 words. If you agree to deliver the first draft in 18 months, that works out at about 1,000 words a week on average. However, behind those words lies a mountain of research and interviews. Depending on the focus of the book, you might need to sift through archives, newspapers, and biographies or even run simulations and analyse data. When researching a topic, it’s worth tracking down the primary source when possible. Just as facts in academic papers can wander astray over the course of several citations [10], well-known quotes and stories may turn out to be apocryphal [11].

It helps to interview people who are involved in the subject area of your book. Your job may work in you favour here: researchers can be happier to talk to a fellow academic than a journalist. However, it’s still important to follow good journalistic practice. Interviews should be on the record, and you should record your conversations to ensure accurate quotes [12]. Researching, interviewing, and writing take a lot of effort, which must be balanced against academic work. Good time management is essential: set aside specific evenings, weekends, and holidays for the book. In my experience, a good writing day will add around 500 to 1,000 words.

## Rule 8: Edit and edit again

Once the initial draft is ready, it will need several rounds of editing. Many of the rules for writing and editing papers [13,14] also apply to popular science books. Avoid long, complex sentences and adverbs. Make sure there is a clear logical structure. Get feedback from colleagues who are not afraid to critique the work. You’ll probably spend most time researching and writing about the topics you don’t know so well, so pay particular attention to sections of the book where you’re on familiar ground; this is where simple errors can creep in. Make sure you also understand what constitutes copyright infringment and libel, especially if you’re covering controversial events [15]. Your publisher should also be able to help advise on this.

When you send the full manuscript to your publisher, there will be two main editing steps. As with any publication, the main editor will advise on broad aspects like content and structure; copyeditors later deal with the grammar and wording. If you’ve signed both a United States and United Kingdom deal, you may also need to ‘translate’ the manuscript for audiences either side of the Atlantic.

## Rule 9: Plan your publicity

Around six months before the publication date, your publisher will start ramping up for the launch. They’ll usually send dozens of review copies of the book to a range of reporters, producers, and editors. They’ll also mail copies to well-known authors and academics to collect ‘blurbs’ (those complimentary quotes you often see on first editions of books). Most publishers have in-house publicists, and they’ll help you pitch book-related articles to newspapers and magazines [16]. These are typically around 700 to 800 words, highlighting stories and ideas from the book that would be of interest to a particular readership.

Very occasionally, a debut book will sell lots of copies without much publicity. However, there seems to be a pretty strong link between sales and traditional publicity efforts. If a book is reviewed or featured in a major newspaper or mentioned on radio or TV, it can shoot up the Amazon rankings.

## Rule 10: Do lots of speaking

Near the book launch date, your publisher can help line up interviews, which may include radio, podcasts, newspapers, and television. If you already have contacts in the media, it’s a good idea to get in touch and see if they’d be interested, too. There may also be the option to give public talks about the book. These might be one-off events or technology- and/or business-focused conferences with several other speakers. Each event will involve slightly different time slots and audiences, so although you may want to include a few recurring topics, you should plan to be flexible.

It can be difficult to avoid publicity conflicting with your scientific work. In some cases, you may need to turn down book-related events because of existing academic commitments. This promotion stage is a lot of work, but it can also be a lot of fun. After months of working alone on the book, it’s an opportunity to share the stories and ideas in your book with a wider audience. As an author, it’s a wonderful feeling to see people enjoy something you've created.

## References

[1] Culture Lab. What popular science books have changed the world? New Scientist 2012 Available from: https://www.newscientist.com/blogs/culturelab/2012/08/what-popular-science-books-changed-the-world.html. Accessed on 10 August 2017.

[2] Kreeger KY. Writing Science. The Scientist 2000. Available from: http://www.the-scientist.com/?articles.view/articleNo/12626/title/Writing-Science/. Accessed on 10 August 2017.

[3] Kucharski AJ. The Perfect Bet: How Science and Math Are Taking the Luck Out of Gambling. 1st ed. Basic Books 2016

[4] Kucharski AJ. The future of medicine In Al-Khalili J. (Ed.), What’s Next? Profile Books 2017

[5] Kucharski AJ. Finding Apollo In Parc S. (Ed.), 50 Visions of Mathematics. Oxford University Press 2014

[6] Bourne PE, Chalupa LM. Ten Simple Rules for Getting Grants. PLoS Comput Biol 2006;2:e12 doi: 10.1371/journal.pcbi.0020012 16501664 10.1371/journal.pcbi.0020012 PMC1378105

[7] Watercutter A. The Rejection Letters That Burned Everyone From George Orwell to Aspiring Clowns. Wired 2014 Available from: https://www.wired.com/2014/01/sundance-free-fail-instagram/. Accessed on 10 August 2017.

[8] Martinez-Condea S, Macknik SL. Finding the plot in science storytelling in hopes of enhancing science communication. Proc Natl Acad Sci USA. 2017;114:8127–8129 doi: 10.1073/pnas.1711790114 28765506 10.1073/pnas.1711790114 PMC5547661

[9] Radford T. A manifesto for the simple scribe–my 25 commandments for journalists. Guardian Online 2011. Available from: https://www.theguardian.com/science/blog/2011/jan/19/manifesto-simple-scribe-commandments-journalists. Accessed on 10 August 2017.

[10] Reich NG, Perl TM, Cummings DAT, Lessler J. Visualizing Clinical Evidence: Citation Networks for the Incubation Periods of Respiratory Viral Infections. PLoS ONE 2011;6:e19496 doi: 10.1371/journal.pone.0019496 21559339 10.1371/journal.pone.0019496 PMC3084881

[11] O'Toole G. Hemingway Didn't Say That: The Truth Behind Familiar Quotations. 1st ed. Little A 2017

[12] Reuters. The Essentials of Reuters sourcing. Available from: http://handbook.reuters.com/?title=The_Essentials_of_Reuters_sourcing. Accessed on 10 August 2017.

[13] Zhang W. Ten Simple Rules for Writing Research Papers. PLoS Comput Biol 2014;10:e1003453 doi: 10.1371/journal.pcbi.1003453 24499936 10.1371/journal.pcbi.1003453 PMC3907284

[14] Weinberger CJ, Evans JA, Allesina S. Ten Simple (Empirical) Rules for Writing Science. PLoS Comput Biol 2015;11:e1004205 doi: 10.1371/journal.pcbi.1004205 25928031 10.1371/journal.pcbi.1004205 PMC4415812

[15] Bingham H. The Writers' and Artists' Yearbook Guide to Getting Published: The Essential Guide for Authors. Bloomsbury 2010

[16] Pitch Database. The Open Notebook 2017. Available from: http://www.theopennotebook.com/pitches/. Accessed on 10 August 2017.

